# Comparison of Faecal versus Rumen Inocula for the Estimation of NDF Digestibility

**DOI:** 10.3390/ani9110928

**Published:** 2019-11-07

**Authors:** Maria Chiaravalli, Luca Rapetti, Andrea Rota Graziosi, Gianluca Galassi, Gianni Matteo Crovetto, Stefania Colombini

**Affiliations:** Dipartimento di Scienze Agrarie e Ambientali, Università degli Studi di Milano, 20133 Milano, Italy; maria.chiaravalli@unimi.it (M.C.); luca.rapetti@unimi.it (L.R.); andrea.rota@unimi.it (A.R.G.); gianluca.galassi@unimi.it (G.G.); matteo.crovetto@unimi.it (G.M.C.)

**Keywords:** NDF digestibility, faecal inoculum, diet composition, in vitro

## Abstract

**Simple Summary:**

The evaluation of fibre digestibility is very important for the formulation of ruminant diets. Fibre digestibility is usually determined in lab with rumen inoculum obtained from cannulated cows. The research of alternative and less invasive inoculum sources is a critical issue that should be addressed. The present study evaluated the potential of faecal inocula, obtained from cows fed different diets, to assess fibre digestibility of different substrates at different incubation times (48, 240 and 360 h). At short incubation time, fibre digestibility obtained with rumen fluid was always higher than those obtained with faecal inocula, confirming a lower activity of the faecal inocula compared with rumen fluid. However, the type of diets fed to the donor animals had a significant effect on fibre digestibility, with a more active faecal inoculum for cows fed a diet based on maize silage. Despite the differences obtained at the short incubation time, the digestibility values at longer intervals showed that faecal inoculum could replace rumen inoculum. As a consequence, faeces may replace rumen fluid as inoculum for end-point measures, avoiding the use of cannulated animals and decreasing the analytical costs.

**Abstract:**

Cow faeces have been investigated as alternative inoculum to replace rumen fluid to determine neutral detergent fibre (NDF) digestibility (NDFD). Aims of this study were to estimate: (1) the NDFD (48 h) of feed ingredients using a rumen inoculum in comparison with faecal inocula from cows fed diets with different forage basis; (2) the undigestible NDF (uNDF) at 240 and 360 h with ruminal fluid and faecal inocula from lactating cows fed two different diets. At 48 h incubation, the NDFD was affected both by feed and type of inoculum (*p* < 0.01) and by their interaction (*p* = 0.03). Overall, the mean NDFD was higher for rumen inoculum than for faecal inocula (585 vs. 389 g/kg NDF, *p* < 0.05), and faecal inoculum obtained from cows fed hay-based diets gave lower NDFD than those from cows fed maize silage (367 vs. 440 g/kg, *p* < 0.05). At long incubation times, the average uNDF was affected by substrate, inoculum and incubation time (*p* < 0.01), but not by their interactions. For each inoculum, significantly lower values were obtained at 360 than at 240 h. Regressions between uNDF with rumen and with the tested faecal inocula resulted in r^2^ ≥ 0.98. Despite the differences at 48 h, the uNDF showed that faecal inoculum could replace rumen fluid at longer incubation times.

## 1. Introduction

Neutral detergent fibre (NDF) concentration of forages varies from 30% to 80% of DM (Dry Matter) with a wide variation for its digestibility [1]: from less than 40% for highly lignified mature legumes to greater than 90% for unlignified immature grass [2]. The accurate estimation of the NDF digestibility (NDFD) is important because ruminant nutritionists and forage plant breeders use in vitro measures of NDFD to assess forage quality, predict diet digestibility, and select plant genotypes for breeding [2]. For these purposes, NDFD is generally determined at short incubation times (for example: 24, 30, 48 h). However, NDFD can also be measured at longer incubation time to estimate the undegradable NDF (uNDF) of feedstuff and, consequently, to predict the potentially digestible NDF (pdNDF) and to estimate the extent of NDF digestion. Moreover, the uNDF content of faeces and total mixed ration (TMR) is used as a marker to estimate in vivo NDF digestibility with a field application at farm scale. 

The in vitro techniques developed to determine NDFD (for example: [3,4]) involve the use of ruminal inoculum. However, in recent time fresh faeces from ruminants have been investigated as alternative inoculum to replace rumen fluid [5,6,7]. Using a fresh faecal inoculum would have some advantages: it is easier to obtain faeces than rumen liquor and rumen cannulated animals are not needed. Faecal inocula from cows have been used to evaluate the in vitro organic matter digestibility (OMD) of forages [6,8] and gas production (GP) of different feeds [5,7]. For example, Akhter et al. [8] obtained significant regression equations between in vitro OMD of eight forages determined with sheep rumen liquor and cow faeces. Another study [6] showed that the OMD of different forages estimated with faecal inoculum was comparable to the OMD determined with cow rumen fluid after 48 h of incubation. Regarding GP, a study [7] showed that in vitro total GP was greater for feeds incubated with cow rumen inoculum as compared to cow faecal inoculum. Mauricio et al. [5] confirmed these results: they observed that cow faecal inoculum gave lower GP volumes and longer lag time than rumen inoculum; however, potential GP was highly correlated between rumen liquor and faecal inoculum. Overall, all the studies indicate that faeces have the potential to be used as inoculum, but some limitations have been identified; one of the most important is the lower enzymatic activity of faecal inocula compared to rumen liquor [5,8]. In this regard, the type of diets fed to the donor animals can change the microbial population within the digestive tract, and therefore in the faeces of animals. For example, Kim et al. [9] observed that the community structure of cow faecal microbiota is greatly affected by diet and it is particularly associated to the dietary forage and concentrate ratio. These changes should be considered in using faeces as microbial inoculum and should be better evaluated. To the best of our knowledge, the effects of diets fed to cows on faecal inoculum activity were never evaluated. Similarly, as far as we know, there are no studies in literature that determined in vitro NDFD with cow faecal inoculum. Overall further research seems needed to fully understand and develop in vitro digestibility techniques using faecal inoculum.

The aims of this study were: (1) to estimate the NDFD (48 h) of several feed ingredients using a rumen inoculum in comparison with faecal inocula obtained both from dry and lactating cows fed diets characterised by a different forage basis; (2) to estimate the uNDF concentrations of different substrates (faeces and feeds) at two incubation times (240 and 360 h) with ruminal fluid and faecal inocula obtained from lactating cows fed two different diets.

## 2. Materials and Methods 

The study was conducted at Cascina Baciocca, the experimental farm of the University of Milan, with the authorisation of the Ministry of Health, authorisation n. 904/2016-PR.

### 2.1. In Vitro Incubations with Faecal and Rumen Inocula at 48 h

The NDFD analyses were conducted on four feeds and using six inocula (five different faecal inocula and one rumen inoculum). The feeds were selected in order to have different NDF contents, as follows: grass hay (533 aNDF g/kg DM), wheat bran (497 aNDF g/kg DM), maize distiller (387 aNDF g/kg DM), and maize silage (373 aNDF g/kg DM). The feeds were dried at 60 °C for 48 h in a forced-air oven and ground to pass a 1-mm Fritsch mill (Fritsch Pulverisette, Idar-Oberstein, Germany). Each sample was weighed (0.250 g) in duplicate in Ankom F57 bags (Ankom Technology, Macedon, NY, USA); two blank Ankom bags (i.e., bags without sample) were also incubated for each inoculum source. Each bag was placed into a pre-warmed 100 mL Erlenmeyer flask closed by a rubber stopper with a Bunsen valve for gas release and maintained at 39 °C in a water bath with constant agitation.

Different faeces were tested as inoculum for a total of five: faeces collected from two cannulated Holstein dry cows fed a diet composed by (g/kg DM) grass hay (700), lucerne hay (130), maize meal (135), soybean meal (30) and vitamin mineral supplement (5), faeces collected from two Holstein lactating cows fed different diets based on the following forages: maize silage (FL-MS), ryegrass and lucerne silages (FL-GLS), wheat and lucerne silages (FL-WLS), and ryegrass and lucerne hays (FL-GLH). Two different donor cows fed the same diet were used for each incubation run. The composition of the lactating cow diets is in Table 1.

All faecal samples were collected immediately after defecation from the floor of the pen and transported in pre-warmed sealed containers to the laboratory for processing. The time lap in faecal collection and processing was within the range of 20 min. Rumen inoculum was collected from the two rumen cannulated donor cows. The rumen fluid was collected before the morning meal and was immediately strained through four layers of cheesecloth into a pre-warmed (39 °C) flask with CO_2_ and mixed with the buffer solution [10] in a 1:2 ratio. Faecal samples were first mixed with the same buffer solution (at the same 1:2 ratio) and maintained for thirty minutes at 39 °C with CO_2_, and then strained through four layers of cheesecloth [5,6,11].

Following the method described by Spanghero et al. [12], 90 mL of each inoculum was dispensed into the Erlenmeyer flasks under anaerobic conditions, flushing the flask with CO_2_. Two runs of incubations were made in a shaking water bath at 39 °C for 48 h. At the end of incubation, the F57 bags were rinsed with cold water until the water ran clear and then placed in a 60 °C forced-air oven to dry. Subsequently, NDF concentration was determined for each bag using the fibre analyser (Ankom Technology, Macedon, NY, USA) and in vitro NDFD was calculated.

### 2.2. In Vitro Incubations with Faecal and Rumen Inocula at 240 and 360 h

The uNDF content was determined on seven substrates using three different inocula (one rumen and two faeces) and two incubation times at 240 and 360 h. 

The substrates were: barley meal (293 aNDF g/kg DM), maize silage (505 aNDF g/kg DM), lucerne hay (538 aNDF g/kg DM), lactating cow TMR (321 aNDF g/kg DM) and the faeces (595 aNDF g/kg DM) produced by a lactating Holstein cow fed the same TMR, grass hay (662 aNDF g/kg DM) and the faeces (753 aNDF g/kg DM) produced by a dry Holstein cow fed the same grass hay. Each substrate was dried at 60 °C for 48 h in a forced-air oven and then ground to pass a 1-mm Fritsch mill (Fritsch Pulverisette, Idar-Oberstein, Germany) Incubations were conducted weighting (0.250 g) duplicate samples in F57 bags and using the DaisyII incubator jars (Ankom Technology, Macedon, NY, USA). Each jar contained four F57 bags for each substrate (two bags for each incubation time) and four F57 blank bags (i.e., bags without sample; two blanks for each incubation time). 

The inocula RD-GH, FL-MS and FL-GLH previously described were tested. Faeces and rumen liquor were collected and treated as described above. The buffer was composed by two solutions as reported by Ankom protocol [13]. Faeces were mixed with the buffer in a ratio of 450 g/L and processed as decribed by Hughes et al. [6], while rumen fluid was added at a dose of 400 mL/jar, using a 1:3 ratio with the buffer [13].

Each inoculum was poured into one pre-warmed jar and two fermentation runs were carried out. Two F57 bags for each substrate and blank were removed at two times: 240 and 360 h. The inocula were renewed at 120 and 240 h replacing the content of jars with a new inoculum. Upon completion of the incubation, jars were emptied and the F57 bags were treated as described above.

### 2.3. Statistical Analysis

All data were analysed by the mixed procedure of SAS (version 9.4) considering the main effects of inoculum, run, substrate and time and their interactions. Data are reported as LS-MEANS.

Linear regressions between the rumen and the faecal inocula at each incubation time were performed by PROC REG procedure of SAS (version 9.4, SAS Institute Inc., Cary, NC, USA).

## 3. Results

### 3.1. NDFD at 48 h of Incubation Using Rumen and Faecal Inocula

The results of NDFD for rumen and faecal inocula at 48 h are presented in Table 2. 

The values of NDFD were affected by inoculum (*p* < 0.01) and by the interaction between inoculum and feed (*p* = 0.03).

Overall, the mean NDFD value was higher for RD-GH (585 g/kg NDF) than for the faecal inocula (389 g/kg NDF, on average) (*p* < 0.05) but there were differences among faecal inocula. Particularly, faecal inocula of cows fed diets based on hays (FD-GH and FL-GLH) resulted, on average, in lower NDFD (367 g/kg NDF) than FL-MS (440 g/kg NDF) (*p* < 0.05). The values obtained using FL-WLS and FL-GLS were intermediate.

Considering each individual feed sample, all feed samples had higher NDFD values using RD-GH treatment (*p* < 0.05), except for maize silage which was not significantly different between RD-GH (498 g/kg) and FL-MS (418 g/kg). The NDFD of maize distillers obtained with FD-GH inoculum was also significantly lower (*p* < 0.05) than the value obtained using FL-MS (383 vs. 534 for FD-GH and FL-MS, respectively). 

### 3.2. Determination of uNDF Using Rumen and Faecal Inocula

The results of uNDF with faecal and rumen inocula at both incubation times (240 and 360 h) are presented in Table 3. 

The average uNDF value was affected by inoculum and incubation time (*p* < 0.01), but not by the interactions between substrate, inoculum and time. Considering the effect of inoculum, the uNDF was not affected by the type of inoculum for RD-GH and FL-GLH; on the contrary, FL-MS had a higher value than RD-GH both at 240 and 360 h, and a higher value at 240 h in comparison with FL-GLH (Table 3). For each inoculum, the time effect was significant with lower values obtained at 360 h (on average, 240 vs. 214 g uNDF/kg DM for 240 and 360 h of incubation, respectively, *p* < 0.01). This time effect has to be ascribed particularly to the faeces samples. Moreover, uNDF decreased more for FL-MS treatment (−15.6%) than for RD-GH (−9.0%) and FL-GLH (−6.8%) from 240 to 360 h of incubation. 

Considering the values for each substrate (Table 3), the dry cow faeces had the highest (*p* < 0.05) uNDF concentration (408 g/kg DM), while the lowest concentrations were obtained for barley meal and lactating cow TMR (74.9 g/kg DM and 83.4 g/kg DM, respectively). Intermediate values (*p* < 0.05) were registered for maize silage (169 g/kg DM), grass hay (234 g/kg DM), lucerne hay (298 g/kg DM) and lactating cows faeces (319 g/kg DM). 

**Figure 1:**
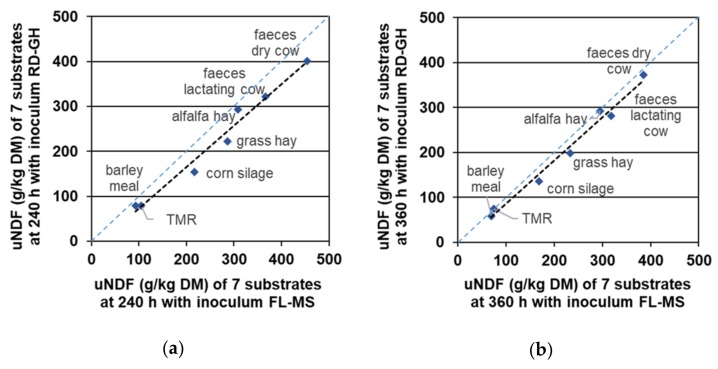
Linear regression between uNDF (g/kg DM) of the tested substrates with the inocula RD-GH (rumen, dry cow—grass hay diet) and FL-MS (faeces, lactating cow—maize silage diet). (**a**) At 240 h, Y = 0.92X (*p* < 0.01) – 19.5 (*p* = 0.35); r^2^ = 0.98, MSE = 443; (**b**) At 360 h, Y = 0.97X (*p* < 0.01) – 11.4 (*p* = 0.44); r^2^ = 0.98, MSE = 276.

**Figure 2:**
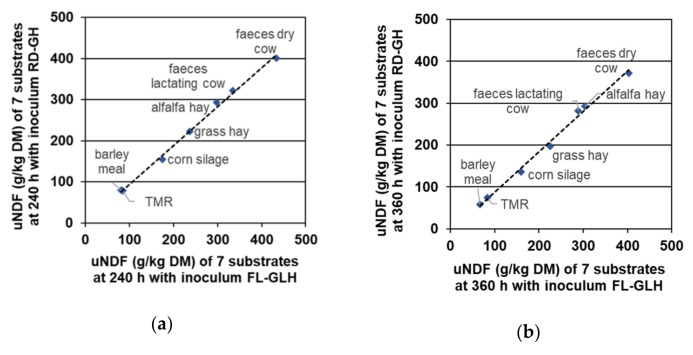
Linear regression between uNDF (g/kg DM) of the tested substrates with the inocula RD-GH (rumen, dry cow—grass hay) and FL-GLH (faeces, lactating cow—ryegrass and lucerne hay diet). (**a**) At 240 h, Y = 0.94X (*p* < 0.01) + 0.03 (*p* = 1.00); r^2^ = 0.995, MSE = 823; (**b**) At 360 h, Y = 0.96X (*p* < 0.01) − 8.68 (*p* = 0.33); r^2^ = 0.99, MSE = 97.0.

All linear regressions (Figure 1 and Figure 2) between uNDF determined with rumen and faecal inocula at 240 and 360 h resulted in high r^2^ values (r^2^ = 0.98 for RD-GH vs. FL-MS at 240 h; r^2^ = 0.996 for RD-GH vs. FL-GLH at 240 h; r^2^ = 0.980 for RD-GH vs. FL-MS at 360 h; r^2^ = 0.994 for RD-GH vs. FL-GLH at 360 h) and slopes within the range 0.92–0.97 showing a high relationship between data.

## 4. Discussion

The objective of this study was to evaluate different faecal inocula (obtained from donor cows fed different diets) to determine NDFD at different incubation times (48, 240 and 360 h). It is well known that in ruminants the intestinal microbes can ferment fibre not digested in the rumen, hence there is the interest to evaluate the potential of faecal inoculum to digest NDF. The evaluation was done on a different set of samples including both forages and fibrous by-products, characterised by a wide variation in NDF digestibility and with an NDF content in the range between 373 and 533 g/kg DM in the first experiment. 

Cow faeces were used in other studies as inoculum source, however, other parameters such as GP [5,7] or in vitro OM digestibility [6,8] were evaluated. The results of these studies underlined that faeces can be used as source of inoculum although the digestibility and the GP determined with faecal inocula were always lower than those found with rumen liquor. Several studies [7,14] showed a consistently longer lag phase with faecal inocula rather than rumen inocula, and this determined lower GP values with faecal inoculum. Similarly, Jiao et al. [15] evaluated fibre digestion in different segments of the post-ruminal tract demonstrating that detectable changes in hydrolysis of NDF occurred after 24 h of in vitro incubation. 

In agreement with the cited studies, the results of the present study showed lower NDFD values at 48 h for cow faecal inocula in comparison with rumen inoculum. The lower NDFD values for faecal inocula were expected since the populations of cellulolytic bacteria are more numerous in rumen inoculum as compared to faecal inoculum [16]. In the rumen, the existence of a greater number of microbial populations is due to the medium stability and various diet compositions, whilst, on the other hand, in the gut nutrients are poor and the nutrient absorption rate is quick [17]. However, Van Vliet et al. [18] showed that diet composition can affect faecal bacterial biomass concentration with highest values associated with high energy diets due to a higher microbial growth in the rumen. Similarly, Lettat et al. [19] showed that increasing the maize silage proportion in the diet reduced the ruminal richness and diversity of the bacterial community but increased the number of total bacteria, which in turn should be reflected in the faecal microbial biomass and hence in the enzymatic activity of inoculum. As far as we know, no studies were conducted to evaluate NDFD using faecal inocula collected from cows fed different diets, although it is known that diet greatly influences the faecal microbiota of cattle [9,20,21]. In the present study FL-MS inoculum significantly affected maize silage NDFD in comparison with the other faecal inocula; it has to be underlined that maize silage was the main ingredient of the diet fed to donor cows of FL-MS inoculum. Although microbial communities were not determined in the present study, it can be speculated that there were some differences in rumen microbial community depending on diet, which in turn affects the faecal microbial community. Lengowski et al. [22] found higher abundance of the cellulolytic species *Fibrobacter succinogenes* during in vitro incubation of maize silage compared to grass silage. Similarly, another study [23] found a larger population of *F. succinogenes* in maize stems compared to other cellulolytic bacteria in in vitro conditions, while Lettat et al. [19] observed higher numbers of *F. succinogenes* in the rumen of dairy cows fed diets high in maize silage compared to those fed diets high in lucerne silage. Maize silage is a C_4_ plant; C_4_ plants usually have a lower nutritive value than C_3_ plants due to a higher proportion of thick-walled cells associated with indigestible fibre in their leaves [24]; therefore, as suggested by Lengowski et al. [25], it may be that *F. succinogenes* has an advantage in degrading maize silage cell walls. The enzymatic system of *F. succinogenes* is more effective at degrading cellulose than the mechanisms used by the other cellulolytic organisms that occupy the same environment [26]. Moreover, *Fibrobacter spp.* have been detected in herbivorous species including the bovine rumen and caecum and in the faeces of different animal species [26]. Overall, it can be speculated that the higher NDFD for FL-MS inoculum can be due both to a higher bacterial concentration and to a possible higher presence of *F. succinogenes* due to the high concentration of maize silage in the diet [19]. 

Despite the difference between rumen and faecal inocula in the NDFD values at 48 h, the incubation conducted at 240 and 360 h showed promising results for faecal inocula to determine uNDF, especially for the inoculum FL-GLH. For all inocula and considering all the substrates, the average uNDF was affected by incubation time with lower values at 360 than 240 h. The uNDF fraction can be estimated by long-term (240 h) in vitro fermentations [27] or by incubating the samples in bags placed in the rumen for 288 h [28]. In the present study, an unconventional incubation time of 360 h (15 days) was also tested in order to compensate for the longer expected lag time of faecal inocula [5]. Unexpectedly, with the in vitro method used in the present study, the incubation time was significant also for the rumen inoculum; with significantly lower uNDF values at 360 than 240 h. Despite the wide application of uNDF, few documented recommendations of the method exist [28]. Recently, Raffrenato et al. [29] indicated that 288 h (12 days) of fermentation were necessary to reach the maximum extent of NDF digestion for the Daisy incubator; hence the incubation time of 240 h used in the present study seems not adequate to evaluate uNDF for some of the incubated substrates (faeces), confirming the results of Raffrenato et al. [29]. 

The effect of the diet fed to donor cows was also significant at long incubation times; however, differently from 48 h incubation, in the long incubation runs the FL-GLH inoculum gave results similar to RD-GH. As suggested by Aiple et al. [30], to obtain an active faecal inoculum, microbial populations in the hind gut should be supplied with fermentable substrate from a varied diet. Although knowledge of the nutrient requirements and supply of the hindgut microbes is limited, it is likely that their requirements are similar to those of the ruminal bacteria [31]. In the present study, cows fed GLH diet (inoculum FL-GLH) had a higher DMI and lower total tract organic matter digestibility (data not presented) than cows fed MS diet. As a consequence, a higher quantity of undigested organic matter was present in FL-GLH inoculum which could have better supported the microbial growth in the long time incubations and positively affecting NDFD. However, it has to be underlined that overall both faecal inocula gave results close to the rumen inocula, as confirmed by the slopes obtained with the linear regression analysis. The long time incubation results are promising since in feed evaluation systems the uNDF is normally computed from the ADL content multiplied by 2.4. However, Raffrenato et al. [32] showed that the ratio between ADL and uNDF is not constant, and, as reported also by Colombini et al. [33], a wrong evaluation of uNDF results in a bias in the prediction of fibre digestibility. Therefore, the use of an appropriate faecal inoculum seems a valuable alternative method to estimate the uNDF avoiding the use of cannulated cows.

## 5. Conclusions

The NDFD results at 48 h obtained with rumen fluid were always higher than those obtained with faecal inocula confirming a lower activity of the faecal inocula compared with rumen fluid. However, the type of diets fed to the donor animals had a significant effect on NDFD values determined by faecal inocula. This should be considered in using faeces as microbial inoculum source. As a prospective view to improve the results at 48 h, the use of fibrolytic enzymes added to the faecal inoculum should be evaluated.

Despite the differences obtained at 48 h, the uNDF results showed that faecal inoculum could replace rumen fluid at longer incubation times. As a consequence, in agreement with the review of Mould et al. [34], faeces may replace rumen fluid as an inoculum for end-point measures. Within the in vitro method applied in the present study, the incubation time of 240 h seems to be not enough to measure uNDF for both faecal and rumen inocula. In the present study, an unconventional time (360 h) was used; hence, further studies seem to be necessary to better define for different substrates (faeces, feeds and TMR) and inoculum the optimum incubation length (in the interval between 240 and 360 h) to evaluate uNDF. 

## Figures and Tables

**Table 1:** Composition of the faecal inoculum donor lactating cow diets (on a dry matter basis)^1^.

Ingredient	MS	GLS	WLS	GLH
(g DM/kg total DM)				
maize silage	493			
maize high moisture ear		286	291	
ryegrass hay	172			253
lucerne silage		268	104	
ryegrass silage		191		
wheat silage			200	
lucerne hay			106	253
grass hay				
soybean meal 44% CP			127	
soybean meal 48% CP	157	81.3		89.8
maize grain ground fine	121	114	127	228
maize grain flaked				86.0
maize gluten feed dry				43.9
wheat bran				11.5
molasses cane	30.3	34.6	19.1	21.5
limestone ground	9.59	8.97	9.11	4.74
sodium bicarbonate	6.15	5.75	5.84	3.16
sodium chloride	3.64	3.40	3.46	2.39
magnesium oxide	3.13	2.93	2.97	1.04
dicalcium phosphate	2.10	1.97	2.00	0.27
smartamine M	0.36	0.30	0.34	0.44
minvit suppl.^2^	1.22	1.14	1.16	1.29

^1^ MS: maize silage diet; GLS: ryegrass and lucerne silage diet; WLS: wheat and lucerne silage diet; GLH: ryegrass and lucerne hay diet; ^2^ minvit composition: vitamin A 400,000 UI/kg, vitamin D3 60,000 UI/kg, vitamin E 1000 mg/kg, vitamin B1 60 mg/kg, vitamin PP 6000 mg/kg, biotin 40 mg/kg, copper sulfate pentahydrate 1900 mg/kg, cupric chelate of amino acids hydrate 4900 mg/kg, calcium iodate anhydrous 40 mg/kg, ferrous carbonate 1800 mg/kg, manganese chelate of amino acids hydrate 9000 mg/kg, sodium selenite 31 mg/kg, zinc oxide 1900 mg/kg, zinc chelate of amino acids hydrate 600 mg/kg, propyl gallate 12 mg/kg, butyl hydroxytoluene 34 mg/kg.

**Table 2:** Effect of inocula^1^ on neutral detergent fibre (NDF) digestibility (NDFD, g/kg NDF) of different feeds at 48 h of incubation.

Sample	RD-GH	FL-MS	FL-GLS	FL-WLS	FL-GLH	FD-GH	*P*	SE
grass hay	578^a^	379^b^	405^b^	411^b^	361^b^	446^b^	0.03	32.4
wheat bran	521^a^	431^b^	425^b^	415^b^	408^b^	407^b^	0.01	18.0
maize distiller	741^a^	534^b^	467^b,c^	441^b,c^	428^b,c^	383^c^	<0.01	34.9
maize silage	498^a^	418^a,b^	288^b,c^	315^b,c^	214^c^	286^b,c^	0.04	50.4
all substrates	585^a^	440^b^	396^b,c^	396^b,c^	353^c^	381^c^	<0.01	14.9

Mean values in a row with different superscripts (a, b, c) differ significantly (*p* < 0.05); ^1^RD-GH: rumen, dry cow—grass hay diet; FL-MS: faeces, lactating cow—maize silage diet; FL-GLS: faeces, lactating cow—ryegrass and lucerne silage diet; FL-WLS: faeces, lactating cow—wheat and lucerne silage diet; FL-GLH: faeces, lactating cow—ryegrass and lucerne hay diet; FD-GH: faeces, dry cow—grass hay diet.

**Table 3:** Effect of inocula^1^ and incubation time on undigestible NDF (uNDF, g/kg DM) of different substrates.

Sample	240 h			360 h			*P*		SE
	RD-GH	FL-MS	FL-GLH	RD-GH	FL-MS	FL-GLH	inoculum	time	
grass hay	223	287	237	198	233	225	0.16	0.15	22.2
lucerne hay	294	309	298	293	295	303	0.62	0.69	9.16
barley meal	79.7	92.9	81.3	58.7	69.8	67.1	0.43	0.03	8.72
maize silage	155	217	175	136	168	160	0.11	0.12	18.3
lactating cow TMR	78.6	104	84.5	74.4	74.9	83.6	0.33	0.13	8.09
faeces dry cow	400	454	434	372	385	403	0.16	0.02	17.0
faeces lactating cow	322	367	334	282	319	289	0.20	0.04	20.6
all substrates	222^b^	262^a^	235^b^	202^c^	221^b^	219^b,c^	<0.01	<0.01	6.06

Mean values in a row with different superscripts (a, b, c) differ significantly (*p* < 0.05); TMR: total mixed ration; ^1^RD-GH: rumen, dry cow—grass hay; FL-MS: faeces, lactating cow—maize silage diet; FL-GLH: faeces, lactating cow—ryegrass and lucerne hay diet.

## References

[1] Bender R.W., Cook D.E., Combs D.K. (2016). Comparison of in situ versus in vitro methods of fiber digestion at 120 and 288 hours to quantify the indigestible neutral detergent fiber fraction of corn silage samples. J. Dairy Sci..

[2] Goeser J.P., Combs D.K. (2009). An alternative method to assess 24-h ruminal in vitro neutral detergent fiber digestibility. J. Dairy Sci..

[3] Hall M.B., Mertens D.R. (2008). In vitro fermentation vessel type and method alter fiber digestibility estimates. J. Dairy Sci..

[4] Lopes F., Ruh K., Combs D.K. (2015). Validation of an approach to predict total-tract fiber digestibility using a standardized in vitro technique for different diets fed to high-producing dairy cows. J. Dairy Sci..

[5] Mauricio R.M., Owen E., Mould F.L., Givens I., Theodorou M.K., France J., Davis D.R., Dhanoa M.S. (2001). Comparison of bovine rumen liquor and bovine faeces as inoculum for an in vitro gas production technique for evaluating forages. Anim. Feed Sci. Technol..

[6] Hughes M., Mlambo V., Lallo C.H.O., Jennings P.G.A. (2012). Potency of microbial inocula from bovine faeces and rumen fluid for in vitro digestion of different tropical forage substrates. Grass Forage Sci..

[7] Ramin M., Lerose D., Tagliapietra F., Huhtanen P. (2015). Comparison of rumen fluid inoculum vs. faecal inoculum on predicted methane production using a fully automated in vitro gas production system. Livest. Sci..

[8] Akhter S., Owen E., Theodorou M.K., Butler E.A., Minson D.J. (1999). Bovine faeces as a source of micro-organisms for the in vitro digestibility assay of forages. Grass Forage Sci..

[9] Kim M., Kim J., Kuehn L.A., Bono J.L., Berry E.D., Kalchayanand N., Freetly H.C., Benson A.K., Wells J.E. (2014). Investigation of bacterial diversity in the feces of cattle fed different diets. J. Anim. Sci..

[10] Menke K.H., Steingass H. (1988). Estimation of the energetic feed value obtained from chemical analysis and in vitro gas production using rumen fluid. Anim. Res. Dev..

[11] Zicarelli F., Calabro S., Cutrignelli M.I., Infascelli F., Tudisco R., Bovera F., Piccolo V. (2011). In vitro fermentation characteristics of diets with different forage/concentrate ratios: Comparison of rumen and faecal inocula. J. Sci. Food Agric..

[12] Spanghero M., Chiaravalli M., Colombini S., Fabro C., Froldi F., Mason F., Moschini M., Sarnataro C., Schiavon S., Tagliapietra F. (2019). Rumen inoculum collected from cows at slaughter or from a continuous fermenter and preserved in warm, refrigerated, chilled or freeze-dried environments for in vitro tests. Animals.

[13] Ankom Technology (2005). In Vitro True Digestibility Using the DAISY Incubator. http://www.ankom.com/media/documents/IVDMD_0805_D200.pdf.

[14] Pandian C.S., Reddy T.J., Sivaiah K., Blϋmmel M., Ramana Reddy Y.R. (2016). Faecal matter as inoculum for in vitro gas production technique. Anim. Nutr. Feed Technol..

[15] Jiao J., Lu Q., Tan Z., Guan L., Zhou C., Tang S., Han X. (2014). In vitro evaluation of effects of gut region and fiber structure on the intestinal dominant bacterial diversity and functional bacterial species. Anaerobe.

[16] El-Meadaway A., Mir Z., Mir P.S., Zaman M.S., Yanke L.J. (1998). Relative efficacy of inocula from rumen fluid or faecal solution for determining in vitro digestibility and gas production. Can. J. Anim. Sci..

[17] Guzmán M.L., Sager R.L. (2016). Ruminant Fecal Inolucum for In Vitro Feed Digestibility Analysis. https://unsl.academia.edu/RicardoSager.

[18] Van Vliet P.C.J., Reijs J.W., Bloem J., Dijkstra J., De Goede R.G.M. (2007). Effects of cow diet on the microbial community and organic matter and nitrogen content of feces. J. Dairy Sci..

[19] Lettat A., Hassanat F., Benchaar C. (2013). Corn silage in dairy cow diets to reduce ruminal methanogenesis: Effects on the rumen metabolically active microbial communities. J. Dairy Sci..

[20] Callaway T.R., Dowd S.E., Edrington T.S., Anderson R.C., Krueger N., Bauer N., Kononoff P.J., Nisbet D.J. (2010). Evaluation of bacterial diversity in the rumen and feces of cattle fed different levels of dried distillers grains plus solubles using bacterial tag-encoded FLX amplicon pyrosequencing. J. Anim. Sci..

[21] Shanks O.C., Kelty C.A., Archibeque S., Jenkins M., Newton R.J., McLellan S.L., Huse S.M., Sogin M.L. (2011). Community structures of fecal bacteria in cattle from different animal feeding operations. Appl. Environ. Microbiol..

[22] Lengowski M.B., Zuber K.H., Witzig M., Möhring J., Boguhn J., Rodehutscord M. (2016). Changes in rumen microbial community composition during adaption to an in vitro system and the impact of different forages. PLoS ONE.

[23] Kozakai K., Nakamura T., Kobayashi Y., Tanigawa T., Osaka I., Kawamoto S., Hara S. (2007). Effect of mechanical processing of corn silage on in vitro ruminal fermentation, and in situ bacterial colonization and dry matter degradation. Can. J. Anim. Sci..

[24] Wilson J.R. (1994). Cell wall characteristics in relation to forage digestion byruminants. J. Agric. Sci..

[25] Lengowski M.B., Witzig M., Möhring J., Seyfang G.M., Rodehutscord M. (2016). Effects of corn silage and grass silage in ruminant rations on diurnal changes of microbial populations in the rumen of dairy cows. Anaerobe.

[26] McDonald J.E., Rooks D.J., McCarthy A.J. (2012). Methods for the isolation of cellulose-degrading microorganisms. Methods Enzymol..

[27] Palmonari A., Gallo A., Fustini M., Canestrari G., Masoero F., Sniffen C.J., Formigoni A. (2016). Estimation of the indigestible fiber in different forage types. J. Anim. Sci..

[28] Krizsan S.J., Huhtanen P. (2013). Effect of diet composition and incubation time on feed indigestible neutral detergent fiber concentration in dairy cows. J. Dairy Sci..

[29] Raffrenato E., Ross D.A., Van Amburgh M.E. (2018). Development of an in vitro method to determine rumen undigested aNDFom for use in feed evaluation. J. Dairy Sci..

[30] Aiple K.P., Steingass H., Menke K.H. (1992). Suitability of a buffered faecal suspension as the inoculum in the Hohenheim gas test: 1. Modification of the method and its ability in the prediction of organic matter digestibility and metabolizable energy content of ruminant feeds compared with rumen fluid as inoculum. J. Anim. Physiol. Anim. Nutr..

[31] Frey J.C., Pell A.N., Berthiaume R., Lapierre H., Lee S., Ha J.K., Angert E.R. (2010). Comparative studies of microbial populations in the rumen, duodenum, ileum and faeces of lactating dairy cows. J. Appl. Microbiol..

[32] Raffrenato E., Erasmus L.J. (2013). Variability of indigestible NDF in C3 and C4 forages and implications on the resulting feed energy values and potential microbial protein synthesis in dairy cattle. S. Afr. J. Anim. Sci..

[33] Colombini S., Galassi G., Crovetto G.M., Rapetti L. (2012). Milk production, nitrogen balance, and fiber digestibility prediction of corn, whole plant grain sorghum, and forage sorghum silages in the dairy cow. J. Dairy Sci..

[34] Mould F.L., Kliem K.E., Morgan R., Mauricio R.M. (2005). In vitro microbial inoculum: A review of its function and properties. Anim. Feed Sci. Technol..

